# Fetal Tachyarrhythmia - Part I: Diagnosis

**Published:** 2004-07-01

**Authors:** Martijn A Oudijk, Gerard HA Visser, Erik J Meijboom

**Affiliations:** * Department of Obstetrics and Gynecology, University Medical Center Utrecht,The Netherlands; † Division of Pediatric Cardiology, Central Hospital University of Vaud, Lausanne, Switzerland

## Introduction

Fetal tachycardia, first recognized in 1930 by Hyman et al [1], is a condition occurring in approximately 0.4-0.6% of all pregnancies [2]. A subset of these cases with more sustained periods of tachycardia is clinically relevant. The necessity of therapeutic intervention in this condition is still a matter of discussion focused on the natural history of the disease. The spectrum of opinions varies from non-intervention [3-2]   based on reports of deterioration of the fetal condition ultimately ending in significant neurological morbidity based on a number of cases in which the tachycardia subsided spontaneously [6], to aggressive pharmacotherapeutic intervention [9-11], or fetal demise [12-14]. Prenatal treatment through indirect, maternally administered drug therapy seems to be the preference of most centers [15] [21]. This matter will be discussed further in *Fetal Tachyarrhythmia, Part II, Treatment*.

The choice of the specific pharmacotherapeutic agents and the chances on success of therapy depend largely on the type of tachycardia. The determination of the type of tachycardia is therefore of the utmost importance [20] [22], however the available diagnostic armamentarium is limited. The most widely used method of diagnosis of fetal tachycardia, M-mode echocardiography, provides a time related documentation of function of the various cardiac structures. The current subdivision into supraventricular tachycardia (SVT), atrial flutter (AF) and ventricular tachycardia (VT) derived from M-mode echocardiography is not sufficient enough for differentiation according to the electrophysiologic mechanism. Several attempts have been made in the last years to increase the accuracy and reliability of M-mode echocardiography by measurement of atrioventricular (AV) and ventriculoatrial (VA) intervals [23,24],   and the addition of Doppler echocardiography [25,26]. New methods registering the actual electrophysiologic events are in development including noninvasive techniques such as magnetocardiography(MCG) [27]. Characteristics of the most common types of fetal tachycardia are described and examples of M-mode echocardiography and FMCG are presented.

## Definitions of tachycardia

The normal fetal heart rate ranges are approximately 120-160 bpm at 30 weeks and 110-150 bpm at term [28,29]. Frequencies up to 170 bpm are considered mildly abnormal, whereas overt tachycardia is usually defined as a heart rate exceeding 170 bpm [28] or 180 bpm [22]. These rhythm abnormalities of the fetus are usually noticed at routine prenatal visits. Fetal echocardiography is used to exclude structural cardiac defects and used to position the M-mode sampling line to intercept both the atrial and ventricular walls. The relationship between peak atrial and ventricular systolic excursions currently provides a division into SVT, AF and VT.

Fetal magnetocardiography, a technique recording the magnetic field generated by the electrical activity of the fetal heart [27,30], offers a more precise delineation of the fetal electrophysiology. Detection occurs non-invasively by sensors cooled by liquid helium positioned several centimeters above the maternal abdomen in a magnetically shielded room. As the maternal heart generates magnetic activity as well, a maternal ECG is recorded simultaneously and substracted from the fetal MCG. This way, an averaged one lead fetal MCG is obtained and allows for a more detailed differentiation of the type of tachycardia.

##  Premature Atrial Contractions

**Figure 1:**
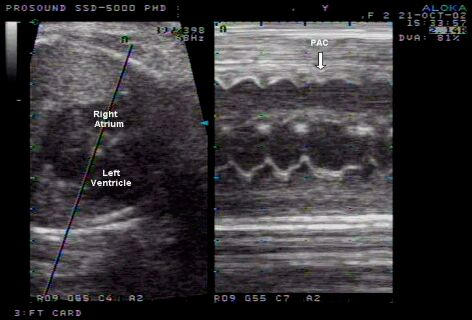
M-mode echocardiography of a premature atrial contraction (PAC)

Premature atrial contractions (PAC’s) do not qualify as a form of fetal tachycardia, and are associated with good outcome. However, in approximately 0.4 % of cases, it may progress to runs of tachycardia and even become persistent. It is therefore recommended that these patients are monitored weekly by doptone to exclude the presence of runs of tachycardia [12,16]. M-mode echocardiography will show premature atrial contractions not followed by a ventricular contraction and a subsequent ‘drop’ in heart rate (Figure 1).

**Figure 2:**
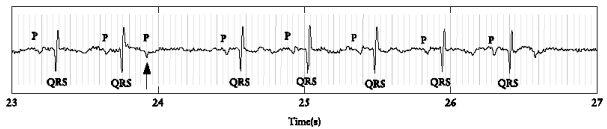
FMCG of a PAC (black arrow)

 The FMCG will show a premature P-wave not followed by a QRS complex (Figure 2).

##  Atrial Flutter

**Figure 3:**
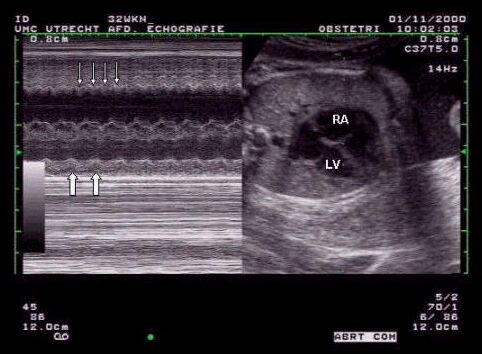
M-mode echocardiography of AF. Flutter contractions of the atrium are indicated by the small arrows; atrioventricular conduction is 2:1 and ventricular contractions are indicated by the large arrows.

AF is a condition that accounts for approximately 21 – 50 % of fetal tachycardia and may be associated with structural abnormalities [14,21,31]. It is defined as an atrial rate ranging from 250 up to 500 bpm with a fixed or variable AV block, as the AV-node is not able to conduct every contraction of the atrium which results in a 2:1 or 3:1 conduction to the ventricles. Rarely in some cases paroxysmal 1:1 conduction is seen. It may be either paroxysmal or incessant in nature and is reported to be associated with fetal hydrops in 7 – 43 % of cases [14,18,31,32]. The most common electrophysiologic mechanism in AF is a re-entry circuit confined to the atrium. M-mode echocardiography will show typical atrial contractions that are followed by ventricular contractions every 2 or 3 atrial contractions (Fig. 3).

**Figure 4.1:**
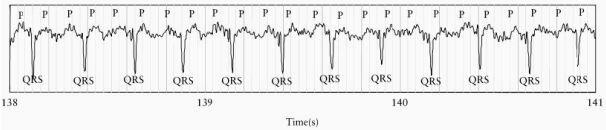
4.1: Raw FMCG trace of atrial flutter with 2:1 conduction.

 FMCG will show typical flutter waves (P-waves) followed by QRS complexes every 2-3 P-waves (Figure 4.1).

## Supraventricular tachycardia

SVT is reported to account for 47 – 68 % of cases of fetal tachycardia [14,18,21] and is associated with a low percentage of structural abnormalities in 2 % of cases. It is defined by a 1:1 atrioventricular conduction in which the atrial contraction precedes the ventricular contraction. Heart rates in SVT most commonly range from 200-300 bpm, is either paroxysmal or incessant in nature and associated with fetal hydrops in 36 - 64 % [14,33]. The condition has several different underlying electrophysiologic mechanisms, such as re-entry using an accessory atrioventricular connection (AVRT), which may be either apparent or concealed. Other possibilities include primary atrial tachycardias and re-entry within the AV node [34].

**Figure 5.1:**
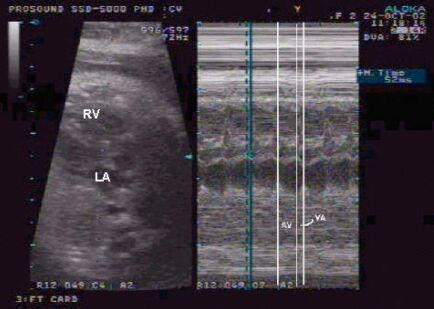
M-mode echocardiography of a SVT with a short VA interval. First white line placed on the peak excursion of the left atrium wall, second line placed on the peak excursion of the right ventricle wall, third line on the consecutive peak atrial wall excursion. The AV interval is markedly longer than the VA interval.

**Figure 5.2:**
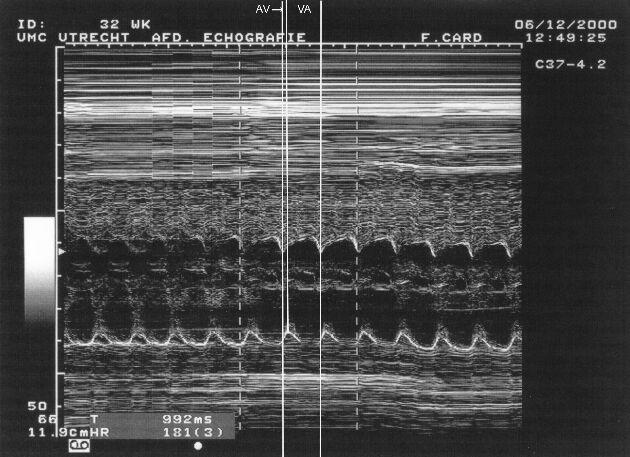
M-mode echocardiography of a SVT with a long VA interval

The diagnosis of these specific types of fetal tachycardia is difficult, if not impossible, with the most widely used current technology, M-mode echocardiography. As mentioned in the introduction, several studies have been conducted to allow for a more specified diagnosis using Doppler and M-mode echocardiography. These techniques are based on the relationship in time between atrial and ventricular wall excursions on M-mode, and flow patterns over the AV and semilunar valve orifices. In most re-entrant tachycardias, conduction through the accessory connection is fast, as in the Wolff-Parkinson-White syndrome (WPW). This results in a short RP interval on the postnatal ECG, comparable to a short VA interval on M-mode (Figure 5.1). In several patients, slow conduction is observed in the accessory pathway, as in Persistent Junctional Reciprocating Tachycardia (PJRT) and Atrial Ectopic Tachycardia (AET) [26], resulting in a long RP interval on the ECG, comparable to a long VA interval on M-mode echocardiography (Figure 5.2). In the literature there has been one case diagnosed as Junctional Ectopic Tachycardia (JET) by Doppler echocardiography [35]. It is extremely rare and we have not encountered JET in the prenatal situation at our center.

**Figure 6:**
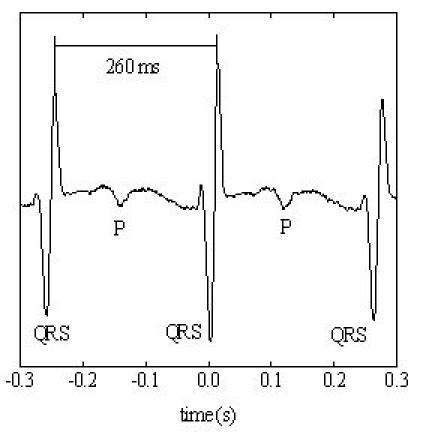
FMCG of a fetal SVT

The detailed electrophysiological events however, are irretrievable by M-mode echocardiography. Specific types of fetal tachycardia have been detected and published using FMCG [36,37]. A more detailed configuration, comparable to the postnatal ECG is obtained. An example of this category of fetal SVT’s is shown in Figure 6.

The electrophysiologic events are clearly defined in this situation, however, a precise diagnosis is still debatable. The SVT shown in Figure 6 for instance, may be AVRT using a concealed accessory pathway, but could also be attributed to represent a PJRT.

## Ventricular Tachycardia

**Figure 7:**
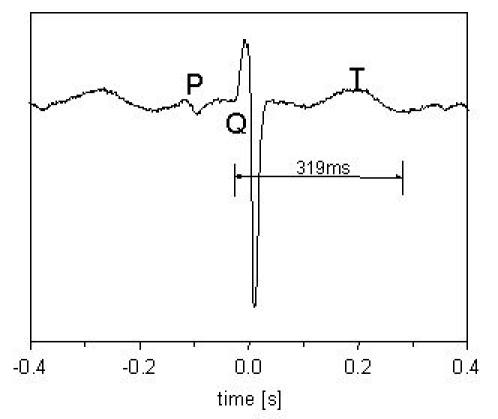
The QT interval is 319 ms, RR interval is 430 ms, QTc interval is 486 ms

VT in the prenatal situation is rare and has only been reported occasionally in the international literature [5,16]. It is usually paroxysmal in nature and outcome depends on the electrophysiologic mechanism. Of the utmost importance is the diagnosis of the congenital Long QT syndrome (LQTS). The origin of this arrhythmia lies in malfunctioning myocardial ion channels as a result of mutations in genes encoding these ion channels. It is characterised by prolongation of the QT interval and the occurrence of polymorphic ventricular arrhythmia (such as Torsade de Pointes). Patients with the LQTS are predisposed to ventricular fibrillation and sudden death [38,39]. The prenatal diagnosis is focused on fetuses of mothers with prolonged QT syndrome to document the possible presence of this syndrome in the fetus. In addition, fetuses presenting with (baseline) bradycardia or intermittent ventricular tachycardia of the torsade de pointe type, the diagnosis of LQTS must be considered as well, as spontaneous mutations have been reported. The suspicion of fetal LQTS may be raised in case M-mode echocardiography shows (baseline) bradycardia varying from 60-110 bpm and intermittent ventricular tachycardia in which there is atrioventricular dissociation. FMCG shows a prolonged QT interval (Figure 7), and the possible presence of intermittent VT [40,;41].

## Conclusions

Fetal tachycardia is a rare disorder, in which difficulties are encountered in the diagnosis of the exact underlying electrophysiological mechanism and is therefore probably underreported. The spectrum of pathologic symptomatology varies from an infrequent paroxysmal tachycardia to a persistent form, which may deteriorate in fetal hydrops, neurological morbidity and even fetal demise. The presence of fetal tachycardia therefore deserves more attention and requires a specialized evaluation to define its electrophysiologic diagnosis.

Fetal atrial flutter is a frequently encountered form of fetal tachycardia and can be well diagnosed by either M-mode echocardiography or FMCG. Fetal SVT remains the most complicated form of fetal tachycardia, both in diagnosis as in treatment. The difficulty in delineating an accurate diagnosis of the specific form and the relative therapy resistance of the various subtypes of this tachycardia, for instance PJRT, makes the development of an optimal treatment protocol complicated.

The most infrequent encountered form of tachycardia is fetal VT, however, if present, it has very serious implications for the fetus. In utero management is complicated and yields a limited success but it is also predictive for an uncertain future after birth. Improved prenatal diagnostic techniques may support a better understanding of the pathophysiology, but does not necessarily result in an improved outcome. Studies on these new techniques will hopefully result in the ultimate goal of reduced morbidity and mortality in the field of fetal tachycardia.

## Figures and Tables

**Figure 4.2:**
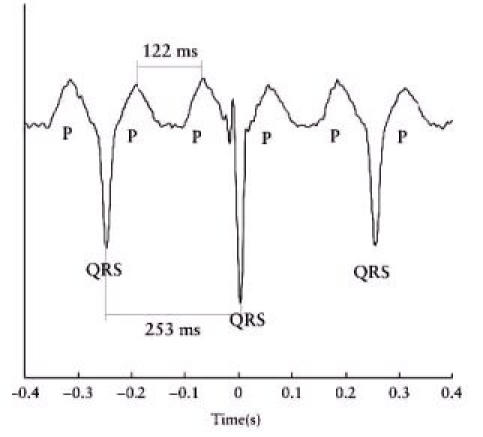
Averaged FMCG trace of atrial flutter with 2:1 conduction.
